# Time-of-Day Variations and Effects of Total Sleep Deprivation on Attention Assessed with the Attentional Demands Task

**DOI:** 10.3390/clockssleep8030041

**Published:** 2026-07-02

**Authors:** Ilaria Di Pompeo, Martina Marcaccio, Giorgia Capitani, Assunta Pompili, Simone Migliore, Giuseppe Curcio

**Affiliations:** Department of Biotechnological and Applied Clinical Sciences, University of L’Aquila, Via Vetoio-Ed.De Meis, 67010 L’Aquila, Italy; ilaria.dipompeo@graduate.univaq.it (I.D.P.); martina.marcaccio@graduate.univaq.it (M.M.); giorgia.capitani@graduate.univaq.it (G.C.); assunta.pompili@univaq.it (A.P.); giuseppe.curcio@univaq.it (G.C.)

**Keywords:** diurnal fluctuations, sleep deprivation, selective attention, divided attention, task switching, cognitive flexibility

## Abstract

Circadian rhythms, sleep homeostasis, and chronotype interact to modulate cognitive processes such as attention and executive functions, leading to fluctuations in performance throughout the day and significant impairments under conditions of misalignment or sleep deprivation. The study was conducted in two phases and included the assessment of subjective measures of sleepiness, vigor, affect, sleep quality, and individual chronotype. In the first phase, 32 healthy participants (19 females; mean age = 24.9 ± 3.14) were tested at four different times of the day to examine diurnal variations in attentional performance. In the second phase, a subsample of 8 participants (4 females; mean age = 24.4 ± 2.20) underwent a total sleep-deprivation protocol and completed the AD-Task over two consecutive days to evaluate the impact of prolonged wakefulness on attentional and executive functioning. In the first phase, results revealed a progressive decline in selective attention across the day (F_(3.93)_ = 3.188; *p* = 0.027; η^2^p = 0.093) and significantly slower reaction times in divided attention during the morning (F_(3.93)_ = 7.3134; *p* < 0.001; η^2^p = 0.191), indicating a time-of-day modulation of attentional resources. In the second phase, total sleep deprivation led to a marked impairment in performance, reflected by slower reaction times (F_(11.77)_ = 1.948; *p* = 0.046; η^2^p = 0.218) and reduced discriminative ability (d′; F_(11.77)_ = 13.438; *p* < 0.001; η^2^p = 0.657), particularly in task-switching conditions. These findings suggest increased vulnerability of executive functions under sleep deprivation, likely associated with reduced efficiency of prefrontal cortical processes. The results confirm that time of the day and pressure of sleep significantly affect attention and cognitive flexibility and further affirm the validity of the AD-Task as a reliable instrument for detecting these variations under ecologically relevant conditions.

## 1. Introduction

Circadian variations, chronotype, and sleep pressure influence cognitive functioning in healthy individuals [1]. Life on Earth has evolved under the influence of environmental cycles such as the light–dark cycle and the seasons, which have led to the development of internal oscillations in organisms, known as biological rhythms, including circadian rhythms lasting approximately 24 h [2,3]. The suprachiasmatic nucleus (SCN) acts as the brain’s master clock, synchronizing physiological functions and regulating variables such as body temperature, hormone secretion, and the sleep–wake cycle [4,5]. Circadian rhythms are also crucial for regulating cognitive processes, including attention, working memory, and executive functions [6,7,8]. Research has shown that cognitive performance varies throughout the day, with peaks generally observed during the day and significant declines at night [7], suggesting the importance of these rhythms in optimizing daily activities [1,9,10]. Disruption of circadian rhythms can cause significant cognitive deficits, particularly in attention and alertness [11]. For example, circadian misalignment, such as that occurring during jet lag or shift work, can lead to reduced attention and increased fatigue [12,13]. Human cognitive performance is regulated not only by circadian mechanisms, which follow an approximately 24 h cycle, but also by homeostatic mechanisms. The circadian rhythm promotes alertness during the day and sleepiness during the night. physiologically influencing body temperature and brain activity. Homeostatic mechanisms, on the other hand, ensure the balance of physiological functions, with alertness occurring in the morning after a good night’s sleep and gradually decreasing over the day as the need for sleep increases [14,15]. Borbély’s [14] two-process model explains the mechanisms that regulate the sleep–wake cycle through the interaction between the S-process and the C-process. The S-process represents the homeostatic pressure of sleep, which increases during wakefulness and dissipates during sleep, especially in the non-REM phases. While the C process is a 24 h circadian rhythm that regulates sleep propensity independently of activity, synchronizing with the light–dark cycle. The propensity to sleep reaches its minimum in the early evening hours, when homeostatic pressure (S) is high, and its maximum in the early morning hours. when homeostatic pressure is low. Both processes interact, influencing the alternation between states of drowsiness and vigilance [1,16,17]. Another aspect to consider when studying circadian and homeostatic effects on cognitive performance is the chronotype, understood as an individual’s natural preference for morning or evening activities. Research suggests that cognitive performance, including attention, is influenced by the alignment between an individual’s chronotype and their daily activities [18]. Indeed, the timing of cognitive activities about an individual’s circadian rhythm can significantly influence performance results. In support of this hypothesis, Salehinejad and colleagues [18] found that cortical excitability, associated with attention functioning, can vary by chronotype and time of day, suggesting that individuals perform better when their activities align with circadian rhythms [18]. The interaction between cognitive ability and chronotype, and misalignment may lead to reduced performance in attention-related tasks [19]. A further phenomenon that may explain the relationship between cognitive performance and chronotype lies in the relationship with mood, whereby morning chronotypes generally report better mood correlated with better attention and cognitive function [20], whereas evening chronotypes may experience greater mood fluctuations resulting in greater attention difficulties [21].

Research has shown that variations in circadian rhythm can also alter the ability to task-switch, thereby affecting performance and speed of execution. Steinborn et al. [22] showed that task difficulty and circadian phase can interact significantly, having a greater impact on performance when individuals must switch between tasks rather than repeat the same task. One explanation for this phenomenon may be related to cognitive flexibility, crucial for task switching, which seems to be more associated with the circadian phase than with sleepiness (a component of a homeostatic nature), indicating that circadian rhythm plays a key role in the ability to adapt to new tasks [23]. Another executive function fundamental to task-switching is inhibitory control, which, in the same way as cognitive flexibility, is subject to impairments in gating the circadian rhythm [24]. Further considerations on homeostatic and circadian influences on cognitive performance come from sleep deprivation studies. Sleep deprivation, whether acute or chronic, significantly impairs cognitive processes such as attention and executive functions; it has been shown that even after just 24 h of sleep deprivation, there is a marked decline in performance on tasks requiring attention [25]. Similarly, chronic sleep restriction (e.g., 4–6 h of sleep per night for about 14 days) can cause a decline in attention comparable to the effects of one night of total deprivation [26]. In support of these considerations, Lo and colleagues [27] introduced the ‘neuropsychological hypothesis’, which suggests that sleep deprivation may cause temporary functional lesions in areas crucial to cognitive tasks, such as the frontal and prefrontal areas. Indeed, we know from other studies that vigilant attention is significantly impaired under sleep deprivation conditions [28]. Different components of attention are affected in different ways by sleep deprivation [29]. Cunningham and colleagues [30] show how sustained focus is significantly impaired, while selective attention may remain more resilient. The mechanisms underlying these deficits are caused by changes in brain function and connectivity, with altered cortical activation during attentional tasks leading to a decreased brain response to visual stimuli and reduced attentional processes [31]. The detrimental effects of sleep deprivation exacerbate as attentional demands increase; indeed, when attention is divided, individuals often struggle to maintain focus on primary tasks, resulting in poorer performance in memory retention and task performance [32,33]. Sleep deprivation has also been shown in some studies to alter the brain’s ability to efficiently process information under divided attention conditions [34]. This phenomenon is particularly evident under multitasking conditions, where cognitive load increases and further stresses attentional resources [35]. Task switching, understood as flexible switching between different tasks, relies heavily on the prefrontal cortex (PFC), a region particularly vulnerable to sleep deprivation [27]. Recent studies show that a single night of sleep deprivation can increase performance decrements in task switching compared to task repetition, so-called ‘switching costs’, impairing the ability to adapt quickly to new demands [36]. These effects may be due to the negative impact of sleep deprivation on cognitive flexibility, which is crucial to performing task-switching effectively [37]. These effects are further amplified in situations where divided attention is required, where overloaded cognitive resources and the ability to handle multiple tasks simultaneously are impaired [32,33], leading to impairments in memory retrieval processes and worsening the ability to adapt to fast-moving tasks [35,38], while also altering the brain’s ability to allocate cognitive resources efficiently [39].

Considering these results, this study aims to investigate the effects of diurnal fluctuations and sleep deprivation on attention, focusing on two fundamental components: selective attention and divided attention, and the switching between them. In contrast to the literature, in this work, a single instrument, the Attentional Demands Task, will be used to study both attentional components while also assessing the switching costs of using them alternately. By observing how time-of-day variations and sleep deprivation modulate attention and influence task-switching performance, this study will contribute to clarifying how circadian and sleep-related factors impact cognitive flexibility and executive functions.

## 2. Results

### 2.1. Descriptives

In the first study, the 32 participants (19F age m: 24.9 ± 3.14) had mean schooling of 14.9 ± 1.67, of which 3.1% were unemployed, 3.1% were employed, and 93.8% were students, good sleep quality (PSQI: 4.44 ± 1.98; rSCRAM: 15.2 ± 2.58), an average level of daytime sleepiness (ESS: 6.00 ± 3.23), an intermediate chronotype (rSCRAM: 14.5 ± 3.28), and mean levels of depressed mood (rSCRAM: 9.94 ± 3.66) and anxious mood (12.9 ± 4.94). The indices of sleep quality and restorative sleep remained stable during the monitoring days; details are shown in the Supplementary Materials.

In the second study, the final sample consisted of eight subjects (4F; age m: 24.4 ± 2.20) with an average schooling of 14.6 ± 2.33, including 12.5% unemployed, 12.5% employed, and 75% students. The participants reported good sleep quality (PSQI: 3.38 ± 1.69; rSCRAM: 16.6 ± 1.60), low levels of insomnia (ISI: 0.5 ± 0.4), an average level of daytime sleepiness (ESS: 5.25 ± 2.49), an intermediate chronotype (rSCRAM: 14.6 ± 3.02), and average levels of both depressed mood (rSCRAM: 9.88 ± 0.9) and anxious mood (10.50 ± 5.9). Sleep quality and restorativeness remained stable over the monitoring days, as shown in the Supplementary Materials. The data from the extracted and analyzed actigraphs are shown in Table 1.

### 2.2. Variations in Subjective Ratings of Sleepiness, Vigor, and Affect Throughout the Day

**Scheme 1:**
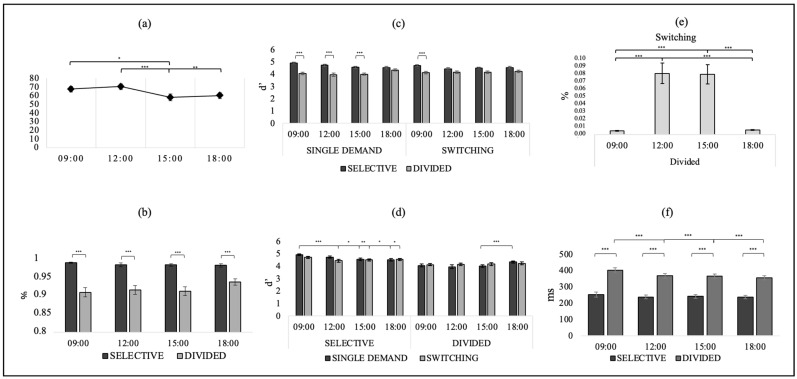
(**a**) Diurnal fluctuations in perceived physical vigor measured by GVAS at different times of the day. (**b**) Interaction between Time and Type of Attentional Demand on HIT RATE performance in the AD-Task. (**c**) Interaction effect of time. attentional demand (selective vs. divided), and task condition (single demand vs. switching) on sensitivity. (**d**) Changes in the ability to discriminate target stimuli (sensitivity, d’) over the day under conditions of single and switched attention for selective and divided attention. (**e**) Fluctuations in FA RATE during the day, depending on the type of attentional demand (selective or divided) and task condition (single or switching). (**f**) Effects of time of day on reaction time in selective and divided attention tasks. All values in the six sections of Scheme 1 are reported as mean ± standard deviation (SD). * *p* < 0.05; ** *p* < 0.01; *** *p* < 0.001.

Analyses performed on the subjective sleepiness index measured by the Karolinska Sleepiness Scale (KSS) showed no significant changes throughout the day, so sleepiness, at least from a perceptual point of view, tends not to vary. The subject perceives himself to be at the same level of sleepiness. About the indices of vigor and affect measured by GVAS, a significant effect of TIME on vigor was found (F_(3.66)_ = 5.82, η^2^p = 0.209, *p* = 0.001). From the post hoc findings, the subject perceived himself to be less physically efficient in the afternoon compared to midday when there was a peak in physical strength (15:00-t_(22.0)_ = 4.096, Mdif = 12.02, *p* = 0.003; 18:00-t_(22.0)_ = 3.991, Mdif = 10.53, *p* = 0.003). For details, see Scheme 1a.

### 2.3. Diurnal Fluctuations in Performance and Speed Indices

Regarding the success rate index in performance at the AD-Task (HIT RATE), an interaction effect emerged between TIME and TYPE OF ATTENTIONAL DEMAND (F_(3.93)_ = 3.403, η^2^p = 0.099, *p* = 0.021), suggesting that attentional demand type influences HIT RATE over time. Post hoc comparisons showed selective attention showed better performance than divided attention at all times investigated: at 09:00 (t_(31.0)_ = 6.5305, Mdif = 0.08061, *p* < 0.001), at noon (t_(31.0)_ = 6.4985, Mdif = 0.06786, *p* < 0.001), at 15:00 (t_(31.0)_ = 6.3736, Mdif = 0.0709, *p* < 0.001), and at 18:00 (t_(31.0)_ = 5.8109, Mdif = 0.04451, *p* < 0.001). This suggests that participants were more accurate when focusing on a single stimulus than when dividing their attention over multiple stimuli; see Scheme 1b.

In general, selective attention shows better results than divided attention at any time of the day.

Regarding the repeated-measures ANOVA applied on the index of discrimination of the target stimulus from the non-target (sensitivity or d’), an interaction effect emerged between TIME * CONDITION * TYPE OF ATTENTIONAL DEMAND (F_(3.93)_ = 3.188, *p* = 0.027, η^2^p = 0.093), which suggests that the effect of time on sensitivity (d’) varies depending on whether participants are engaged in selective or divided attention tasks, in the single demand or switching condition. The post hoc findings show that selective and divided attention exhibit two distinct behaviors in the variability of the ability to recognize the target stimulus. In selective attention, compared to performance in the 09:00 ‘SINGLE DEMAND’ condition, there appears to be an increase in the difference between single demand and switching starting at noon (t_(31.0)_ = 5.8359, Mdif = 0.48092, *p* < 0.001), with a worsening of switching. Over the day, the target stimulus discrimination index worsens both in the single demand condition at 15:00 (t_(31.0)_ = 4.2938, Mdif = 0.35867, *p* = 0.012) and 18:00 (t_(31.0)_ = 4.0025, Mdif = 0.39265, *p* = 0.025) than in the switching condition at the same times (15:00-t_(31.0)_ = 5.1205, Mdif = 0.41372, *p* = 0.001; 18:00-t_(31.0)_ = 4.4619, Mdif = 0.37820, *p* = 0.008) than in the morning. In divided attention, however, the ability to recognize the target stimulus remains stable, showing only one statistically significant difference in afternoon performance in the single demand condition (t_(31.0)_ = 3.7760, Mdif = 0.32747, *p* = 0.044), highlighting how better performance is achieved in the late afternoon than in the postprandial phase. Details are shown in Scheme 1c.

The TIME * CONDITION * TYPE OF ATTENTIONAL DEMAND interaction effect also shows that the difference between the two attentional components (selective and divided) remains marked mainly in the single condition until the early afternoon. In fact, from the post hoc, it is evident that selective attention predicts a better ability to recognize the target stimulus among the irrelevant ones at 09:00 a.m. (t_(31.0)_ = 8.3737, Mdif = 0.85939, *p* < 0.001), at noon (t_(31.0)_ = 5.2615, Mdif = 0.76653, *p* < 0.001), and in the postprandial phase (t_(31.0)_ = 5.3059, Mdif = 0.54929, *p* < 0.001); in the late afternoon, on the other hand, the two attentional demands tend to equalize. In the switching condition, however, the difference between the two types of attention is evident only at 09:00 a.m. (t_(31.0)_ = 5.9530, Mdif = 0.58419, *p* < 0.001). For more details, see Scheme 1d.

About the repeated-measures ANOVA applied to the number of commission errors (FA RATE), an interaction effect emerged between TIME * CONDITION * TYPE OF ATTENTIONAL DEMAND (F_(3.93)_ = 18.1, *p* < 0.001, η^2^p = 0.368), which suggests that the effect of time on the amount of committal errors varies depending on whether participants are engaged in selective or divided attention tasks, in the single or switching demand conditions. Post hoc comparisons showed that divided attention in the switching condition was more sensitive to time-of-day effects, with more commission errors at midday and early afternoon compared to morning and late afternoon. Specifically, errors were higher at 12:00 than in the morning (t_(31)_ = −5.62, Mdiff = −0.077, *p* < 0.001) and late afternoon (t_(31)_ = −5.61, Mdiff = −0.076, *p* < 0.001), and at 15:00 than in the morning (t_(31)_ = −5.90, Mdiff = −0.075, *p* < 0.001) and late afternoon (t_(31)_ = −5.89, Mdiff = −0.074, *p* < 0.001), as shown in Scheme 1e.

Repeated-measures ANOVA applied to the speed of responding to task stimuli (RTs) showed an interaction effect between TIME and TYPE OF ATTENTIONAL DEMAND (F_(3.93)_ = 7.3134, *p* < 0.001, η^2^p = 0.191), which suggests that the effect of time on stimulus responsiveness varies depending on whether participants are engaged in selective or divided attention tasks. Indeed, post hoc comparisons show that in general selective attention predicts better reaction times than divided attention at all times investigated (09:00-t_(31.0)_ = −18.878, Mdif = −148.27, *p* < 0.001; 12:00-t_(31.0)_ = −16.652, Mdif = −129.35, *p* < 0.001; 15:00-t_(31.0)_ = −19.088, Mdif = −123.28, *p* < 0.001; 18:00-t_(31.0)_ = −20.097, Mdif = −118.87, *p* < 0.001). In addition, it is also possible to highlight the effect of time of day on reactivity to respond to task stimuli in divided attention in which 09:00 performance turns out to be the worst compared to the other times (12:00-t_(31.0)_ = 7.223, Mdif = 33.79, *p* < 0.001; 15:00-t_(31.0)_ = 5.022, Mdif = 36.69, *p* < 0.001; 18:00-t_(31.0)_ = 6.471, Mdif = 45.32, *p* < 0.001) being characterized by the most reaction times throughout the day; see Scheme 1f.

### 2.4. Variations in Subjective Ratings of Sleepiness, Vigor, and Affect over the Two Experimental Days

**Scheme 2:**
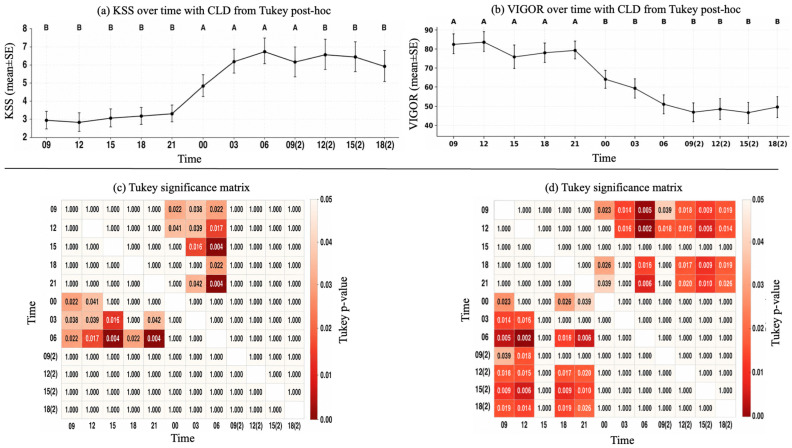
Displays (**a**) subjective sleepiness (KSS) and (**b**) perceived physical efficiency (GVAS_vigor) across 12 time points over two consecutive days, shown as line plots (mean ± SE) with CLD groupings based on Tukey’s post hoc tests (*p* < 0.05). Letters above time points indicate Compact Letter Display (CLD) clusters resulting from Tukey’s post hoc test; time points sharing the same letter show no significant differences (*p* > 0.05), while different letters indicate significant differences (*p* < 0.05). The Tukey post hoc comparison heatmap illustrates statistical differences between time points. Color intensity reflects the magnitude of the *p*-value (darker = more significant; white = not significant). Only pairwise comparisons that were significant in Tukey’s output are displayed, while other cells are set to *p* = 1 to improve interpretability (**c**,**d**).

A repeated-measures ANOVA showed a main effect of TIME on perceived current sleepiness level (F_(11.77)_ = 10.6, *p* < 0.001, η^2^p = 0.602). Post hoc comparisons show that subjects tended to experience greater sleepiness from midnight onwards on the night of sleep deprivation and then maintained this level of sleepiness the following day, as shown in Scheme 2a,c.

A repeated measures ANOVA showed a main effect of TIME on perceived vigor as measured by GVAS (F_(11.77)_ = 17.8, *p* < 0.001, η^2^p = 0.718). Post hoc comparisons show that subjects tended to experience less physical effectiveness starting the night of deprivation and throughout the following day than they experienced on the day before sleep deprivation, see Scheme 2b,d.

No significant changes were observed in the affect scale as measured by GVAS.

### 2.5. Changes in Performance and Speed Indices Assessed by Behavioral Measures as a Function of Sleep Deprivation

**Scheme 3:**
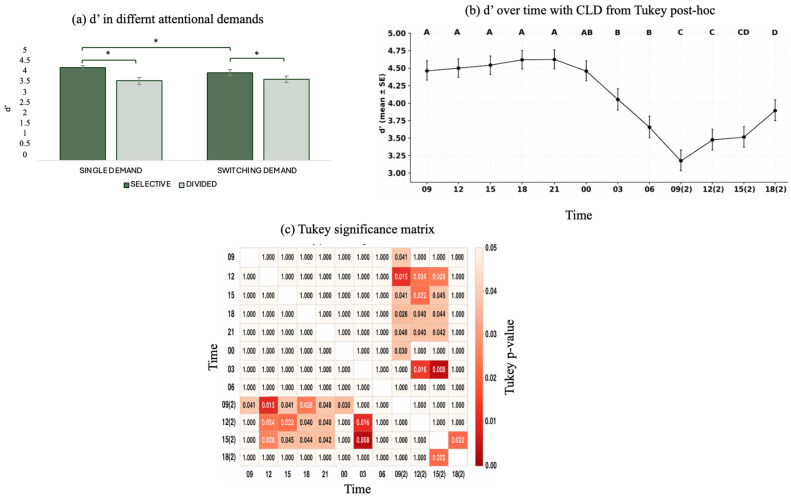
(**a**) Variations in target discrimination ability (sensitivity, *d*′) across the two experimental days under single and switching demand conditions for selective and divided attention tasks. Data represent mean d′ across the 12 time points (mean ± SD). Asterisks indicate levels of statistical significance (* *p* < 0.05). (**b**) Average performance of d′ at 12 time points (mean ± SE). The letters of the Compact Letter Display (CLD) above each point indicate Tukey’s post hoc groupings, where shared letters denote non-significant differences and different letters indicate significant contrasts (*p* < 0.05). The Tukey post hoc comparison heatmap illustrates statistical differences between time points based on d′ values. Color intensity reflects the magnitude of the *p*-value (darker = more significant; white = not significant). Only pairwise comparisons that were significant in Tukey’s output are displayed, while other cells are set to *p* = 1 to improve interpretability (**c**).

The repeated-measures ANOVA performed on the target stimulus discrimination index (d’) showed an interaction effect between CONDITION and TYPE OF ATTENTIONAL DEMAND (F_(1.7)_ = 15.177, η^2^p = 0.684, *p* = 0.006), in which from the post hoc comparisons a better ability to identify the target stimulus among the no-targets in selective attention is found compared to the divided attention in both the single demand (t_(7.0)_ = 5.78, Mdif = 0.6129, *p* = 0.003) and switching (t_(7.0)_ = 3.82, Mdif = 0.3029, *p* = 0.026) conditions. Furthermore, the post hoc comparisons also reveal a statistically significant difference in selective attention between the single demand and switching (t_(7.0)_ = 3.65, Mdif = 0.2306, *p* = 0.032) condition, in which there is a worsening of the target stimulus discrimination index when investigated in switching. In addition, a main effect of TIME (F_(11.77)_ = 13.438, η^2^p = 0.657, *p* < 0.001) was found to show that the passage of hours and increased wakefulness due to the sleep deprivation manipulation affect the ability to detect the target stimulus while ignoring the no-target stimulus. Post hoc findings show that the target stimulus discrimination index tends to decrease at 09:00 on the post-sleep deprivation day and then slowly rises again during the day, becoming non-statistically different at 18:00 compared to the day before the manipulation; see Scheme 3a–c.

**Scheme 4:**
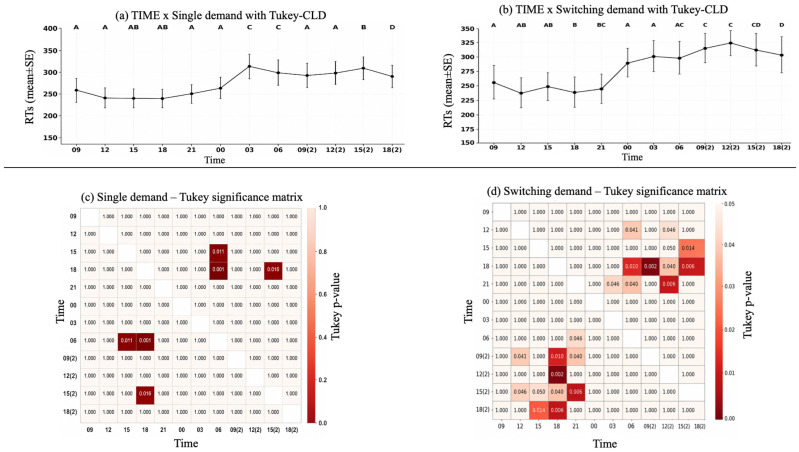
Displays reaction times in the switching (**a**,**c**), and single demand (**b**,**d**) conditions across 12 time points over two consecutive days, shown as line plots (mean ± SE) with CLD groupings based on Tukey’s post hoc tests (*p* < 0.05). Letters above time points indicate Compact Letter Display (CLD) clusters; time points sharing the same letter do not differ significantly, whereas different letters indicate significant differences. The Tukey post hoc comparison heatmaps (**c**,**d**) illustrate pairwise differences between time points. Color intensity reflects the magnitude of the *p*-value (darker = more significant; white = not significant). Only significant Tukey comparisons are displayed, while non-significant cells are set to *p* = 1 for clarity.

The repeated-measures ANOVA performed on the reaction times showed an interaction effect between TIME and CONDITION (F_(11.77)_ = 1.948, η^2^p = 0.218, *p* = 0.046), and post hoc comparisons show that the two conditions (single demand and switching) evolve differently as a function of sleep deprivation. In the single demand condition seems to show greater resistance to sleep deprivation and worsening reaction times in the post-sleep deprivation day at 6 a.m. and 3 p.m. only when compared to the peak times of the previous day; see Scheme 4a,c. On the other hand, in the switching condition, there is a significant increase in reaction times on the post-sleep deprivation day in the early morning and in the early afternoon compared to almost all assessment times of the previous day; see Scheme 4b,d.

**Figure 1:**
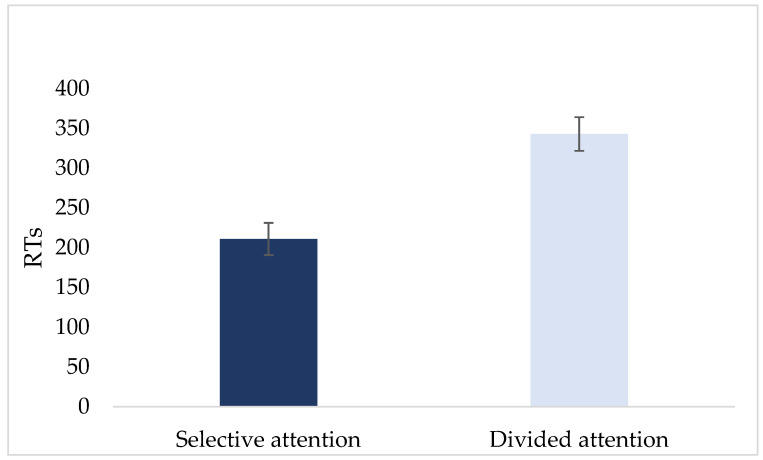
Comparison of Reaction Times Between Selective and Divided Attention Tasks.

In addition, a main effect of TYPE OF ATTENTIONAL DEMAND (F_(1.7)_ = 275.186, η^2^p = 0.975, *p* < 0.001) has been shown, and post hoc comparisons (t_(7.0)_ = −16.6, Mdif = −132, *p* < 0.001) show that selective attention generally predicts shorter reaction times than divided attention, as shown in Figure 1.

## 3. Discussion

This study investigated diurnal fluctuations and the effects of total sleep deprivation on two components of attention and the ability to switch between selective and divided attention. The first phase evaluated how daytime fluctuations influence selective and divided attention and the cognitive cost of switching between them. In the second phase, the impact of a night of total sleep deprivation on the same attentional dimensions was examined. A sample of 32 participants (19 women, average age: 18–30 years) with good sleep quality, no neurological or psychological problems, and an intermediate chronotype was considered to assess the influence of time-of-day on cognitive performance under investigation. During the experimental phase, the participants, selected based on their scores in the Pittsburgh Sleep Quality Index (PSQI) and monitored with the Karolinska Sleep Diary (KSD), completed the subjective questionnaires of sleepiness, vigor, and affection and subsequently performed the Attentional Demands Task (AD-Task) at four different times of the day (09:00, 12:00, 15:00, and 18:00), under controlled conditions and without distractions. The sample of the second phase consisted of eight participants (four women and four men, age 18–30 years), selected based on their scores in the Pittsburgh Sleep Quality Index (PSQI) and monitored throughout the week with the Karolinska Sleep Diary (KSD) and using actigraphs. In this phase, participants were tested for a total of 36 h of continuous waking. After subjectively assessing their sleepiness, vigor, and affectivity using specific questionnaires, they completed the AD-Task every three hours under controlled conditions and without distractions.

The results, taken with caution given the small sample size, seem to come close to the hypothesis that diurnal fluctuations and sleep deprivation significantly modulate attentional performance during the day. This finding could be consistent with the findings of Valdez [10], who showed that selective and divided attention is subject to time-of-day and sleep pressure variations.

Regarding subjective variables, contrary to expectations, subjects did not show significant changes in subjective sleepiness levels, as measured by the KSS scale, during the day without sleep deprivation. This result suggests that, from a perceptual perspective, the participants tend to maintain a constant perception of their alertness. We know from the literature that daytime sleepiness is often associated with poor sleep quality, irregular sleep schedules, and insufficient sleep duration [40,41,42]. Sleep quality is considered a good predictor of daytime sleepiness, often associated with sleep disorders [43]. Age may also contribute to the onset of daytime sleepiness, with the elderly reporting higher levels of sleepiness [44,45]. Furthermore, psychological factors such as anxiety and depression can significantly influence daytime sleepiness and general well-being [46,47]. In our study, the lack of statistically significant differences in current and daytime sleepiness indices is probably associated with the characteristics and small size of the sample analyzed. The young age, good health, good sleep quality, and unremarkable levels of anxiety and depression of the participants in this work appear to be characteristics far removed from those usually associated with increased daytime sleepiness, such as sleep disorders, advanced age, or high levels of psychological distress.

These data may be supported by the accumulation of sleepiness observed during the night of sleep deprivation, which may help support the hypothesis that sleep loss leads to an increase in the perception of sleepiness that persists into the following day. As extensively demonstrated in the literature, sleep deprivation causes a cumulative increase in sleepiness, which is caused by the activation of homeostatic sleep pressure, understood as the body’s natural need to sleep that accumulates the longer one stays awake [48,49]. Several studies have explained that this progressive accumulation of sleepiness may be related to changes in brain activity. Sleep deprivation may alter connectivity between neural networks, impairing vigilance and cognitive abilities [50,51]. Changes in hormones and brain neurochemistry associated with sleep deprivation may also exacerbate sleepiness and subsequent cognitive deficits [52]. It is known that malfunctioning of the thalamic and prefrontal regions correlates with increased perceived sleepiness, as it is crucial for maintaining vigilance [53]. Increased subjective sleepiness leads to reduced cognitive performance, with proven effects in slowing down reactions and increased distraction [54]. Several studies have focused on assessing the relationship between sleepiness and cognitive performance, finding that higher levels of daytime sleepiness may be associated with lower test accuracy and lower scores in cognitive performance [50,55,56].

The analyzed data reveal slight daily fluctuations and some effects of sleep deprivation on perceived physical fitness, measured using the GVAS scale. Regarding fluctuations due to time of day, a peak was observed in the middle of the day, followed by a decrease in the afternoon; this reduction increased with the number of waking hours and was particularly marked in sleep-deprived conditions. This phenomenon, albeit with cautious interpretation, is consistent with what is reported in the literature regarding the homeostatic and circadian processes underlying the postprandial drop in body temperature and alertness [57]. Furthermore, some studies suggest that sleep deprivation may cause reduced physical efficiency through decreased muscle strength, impaired metabolic responses, and loss of cognitive function [58,59,60].

In the behavioral field, the AD-Task proved to be a useful tool for assessing the two attentional processes for which it was created and measuring the cognitive cost of switching from one to the other, providing significant results in the scenarios investigated. Despite the absence of diurnal fluctuations in subjective sleepiness, speed and performance measures obtained on the AD-Task appear to show significant differences between selective attention and divided attention, as well as an interaction with time of day [61,62]. This result can be interpreted thanks to the studies of Liu et al. [50], who highlighted how subjective ratings of sleepiness may sometimes not accurately reflect the real level of cognitive impairment.

Regardless of time of day and sleep deprivation aspects, selective attention is more accurate than divided attention, both in terms of speed and overall performance, confirming the fact that focusing on a single stimulus requires fewer cognitive resources than dividing attention among several stimuli. One explanation may stem from the consideration that repeated exposure to a target may reinforce cognitive responses, making information processing more efficient [63], whereas dividing attention by increasing cognitive load may lead to conflicts between competing stimuli [64]. These considerations are in line with the idea that focusing attention on a single stimulus allows for facilitation in the allocation of cognitive resources, as indicated by the results of Smart and collaborators [65], confirmed by an increased involvement of the P3 component during focused attention.

In support of these considerations, in terms of speed, it was found that reaction times are slower in the morning when performing divided attention tasks, whereas they remain more stable when selective attention is involved. A phenomenon that could explain this result is sleep inertia, understood as a transient state of reduced cognitive performance after waking up that usually results in decreased alertness and cognitive slowdown, ranging from 15 to 120 min after waking up [66,67]. What emerges in our study seems to be confirmed by the literature, in which it is highlighted that selective attention tasks are less susceptible to the negative effects of sleep inertia. Burke and colleagues [68] find that performance in a visual search task known to assess selective attention is less affected by sleep inertia than in tasks requiring divided attention, which are more affected by changes in cognitive state. Sleep deprivation appears to contribute to these results, with a slowing of reaction time extending to both attentional demands and showing a decrease in the morning and postprandial phases of the day following sleep deprivation. Furthermore, in support of what has been discussed so far, this effect is slightly more evident when the two types of attention are studied under switching conditions, confirming that a greater cognitive load impairs performance more [69]. From the literature, we know that the effects of sleep deprivation on attentional concentration vary significantly depending on the time of day, with a particular decrease in the morning [70,71,72] and a tendency to improve throughout the day, with faster response times in the late morning compared to the early morning hours [73]. In some studies, it has been found that an explanation for the worsening of reaction times in the postprandial phase may be related to the fact that food intake and rapid fluctuations in blood glucose levels can interact negatively with sleep deprivation, inducing a decline in the ability to respond promptly to presented stimuli [74,75,76].

As regards a more specific aspect of performance, it should be noted that sensitivity in the response to target stimuli (d’) shows daily fluctuations in a different way depending on the attentional component involved.

Selective attention is more influenced by the time of day, showing evident difficulties in recognizing the stimulus to respond to and in inhibiting others in the afternoon, especially under switching conditions. Selective attention, although benefiting from a lower cognitive load, is more exposed to the costs of task switching, suggesting that its efficiency is related to cognitive flexibility and inhibitory control of workload [77]. In this regard, Monsell [78] argues that the efficiency of inhibitory control is compromised when returning to a more focused attentional mode after performing a divided attention task. This is because divided attention requires a constant balance between multiple information streams, which can leave a residual proactive interference that hinders the transition to a more selective focus [78]. Furthermore, according to Allport et al. [79], simpler tasks tend to be more affected by task switching after a more complex task, as cognitive resources adapt to a higher level of processing and the return to a more selective control requires a less immediate disengagement process. These considerations might explain why selective attention, throughout the day, performs better in its ability to accurately respond to presented targets only when assessed in isolation, away from switching costs. The only time of day when sensitivity is more efficient in selective attention than in divided attention, even under switching conditions, is in the early morning when the possible effects of sleep inertia might influence the more complex attentional component without compromising the simpler one [68].

Overall, time-of-day factors may modulate target stimulus discrimination differently in the two attentional components analyzed. Selective attention appears to be more influenced by diurnal variations, with a noticeable decline in the afternoon, especially during task switching. One explanation for this effect may be that a high level of executive control to filter out irrelevant stimuli, a process known to involve fronto-parietal networks, is usually strongly influenced by cognitive fatigue accumulated during the day [80,81].

Divided attention tends to be less susceptible to diurnal fluctuations, showing only a slight postprandial decline due to the temporary reduction in cognitive resources [82]. The lower propensity to deterioration over the day compared to selective attention may be related to greater task automation or the involvement of separate cognitive resources that reduce the need for active control [80,83]. In general, the afternoon decline is more pronounced in tasks that depend on executive control, whereas divided attention, supported by more automatic processes, is less vulnerable to fatigue, showing only a slight temporary decline after meals, as also shown by the increase in commission errors [84].

The effects of sleep deprivation appear to occur in the analyzed variables with a more consistent course in which deterioration occurs in both attentional capacities, especially when involved in switching.

These phenomena can develop at the performance level, compromising the ability to discriminate the target stimulus (d′), which worsens with increasing waking hours, and at the speed of execution, with increasingly slower reaction times correlated with sleep deprivation. From the literature, we know that sleep deprivation can compromise the prefrontal cortex, a key region for cognitive flexibility and switching between tasks [27,39,85], leading to a deterioration in both attentional demands (selective and divided), with a more pronounced impact on the latter due to the greater cognitive load required [32,39]. The cognitive cost associated with task switching undergoes a non-negligible increase with prolonged wakefulness, with a massive increase in reaction time and a decrease in accuracy in task-switching conditions, confirming that sleep loss can amplify the vulnerability of executive processes [32,36]. Reduced functional connectivity and cortical activation under sleep deprivation, found in other studies, could explain the slowing of reaction time (RT) and reduced accuracy [27]. Furthermore, these functional impairments could result in an alteration of top-down control, which can hinder the management of irrelevant stimuli and the resolution of cognitive conflicts [31]. It follows that switching costs may be more pronounced in cognitively complex tasks due to the greater vulnerability of demanding tasks to sleep loss [79].

## 4. Materials and Methods

### 4.1. Participants

Two separate experiments were conducted. In the first, 32 participants (19 women; mean age = 24.9 ± 3.14 years) were included from an initial sample of 37 to investigate diurnal fluctuations in attention. Five participants were excluded due to technical issues with the equipment. In the second study, which examined the effects of sleep deprivation on attention, a subsample of eight participants (4 women and 4 men; age range: 18–30 years) was included after two participants were excluded due to technical issues with the equipment. In both studies, no one reported neurological or psychological problems, and all had normal vision and hearing. Participants were recruited from the University of L’Aquila.

Both protocols were approved by the Multidisciplinary Internal Review Board of the University of L’Aquila (code 06/2025) and were conducted according to the principles established in the Declaration of Helsinki. Before participation, all individuals provided written informed consent and were informed that they could withdraw from the experiment at any time without consequences.

### 4.2. Instruments

Pittsburgh Sleep Quality Index (PSQI). The PSQI questionnaire [86,87] was used to assess sleep quality in the study sample, with reference to the previous month. It comprises 19 questions and covers seven areas: subjective sleep quality, sleep latency, sleep duration, habitual sleep efficiency, sleep disturbances, use of sleep medications, and daytime dysfunction. Each item is scored from 0 to 3, with an overall score of >5 indicating poor sleep quality. The Italian version demonstrates good internal consistency (Cronbach’s α = 0.83).

Karolinska Sleep Diary (KSD). The characteristics of sleep quality and continuity during the nighttime period of the experiment were recorded using the KSD [88], completed within 30 min of waking to maximize reliability. The diary assesses continuity, depth, number, and duration of awakenings, perception of restfulness, time of final awakening, and contextual factors affecting sleep quality.

Karolinska Sleepiness Scale (KSS). The KSS questionnaire [89] was used to measure the level of subjective momentary sleepiness. It consists of a 9-point scale where 1 equals extremely alert, and 9 equals extremely sleepy, and reflects the psychophysiological state of the previous 10 min. The scale is sensitive to circadian and situational factors and is correlated with EEG and behavioral measurements of sleepiness. It takes about 5 min to complete.

Epworth Sleepiness Scale (ESS). The ESS questionnaire [90] was used to measure habitual daytime sleepiness. This is an eight-question questionnaire that assesses the likelihood of falling asleep in everyday situations. The total score ranges from 0 to 24, with scores ≥ 10 indicating excessive daytime sleepiness.

Global Vigor–Affect Scale (GVAS). Current subjective vigor and affect were measured with the GVAS [91], an eight-item Likert-type scale assessing energy, vitality, and positive/negative mood. Administered immediately before task execution, it provides a psychometric index of acute psychophysiological state.

Insomnia Severity Index (ISI). In the sleep deprivation sub-sample, a questionnaire was also used to assess the severity of perceived insomnia. The ISI [92] includes seven questions on sleep difficulties, satisfaction, interference with daily life, and discomfort, with a score ranging from 0 (none) to 4 (extreme). The total score (0–28) indicates the severity of insomnia, which can be absent, mild, moderate, or severe. The questionnaire takes a few minutes to complete and is a useful tool for both clinical assessment and monitoring improvements over time. Studies have shown that it is a reliable, valid tool that is sensitive to changes with treatment.

The rSCRAM (Revised Sleep, Circadian Rhythms, and Mood) questionnaire is a self-report instrument consisting of 16 items on a 6-point Likert scale (ranging from “Strongly Disagree” to “Strongly Agree”), which assesses four interrelated constructs: sleep quality (Good Sleep), chronotype as diurnal preference (Morningness), depressed mood (Depressed Mood), and anxious mood (Anxious Mood), with 4 items for each subscale. The instrument was developed and validated on an Italian sample, based on the original SCRAM version by Byrne and collaborators [93], with the addition of a subscale for anxiety. It has demonstrated excellent psychometric properties, with satisfactory internal consistency (α = 0.72–0.90) and two-week test–retest reliability (r = 0.73–0.82), as well as good convergent and divergent validity [94].

Actigraphs. In the sub-sample involved in assessing the effects of one night of sleep deprivation. Actiwatch Spectrum Plus (Philips) actigraphs were used to obtain objective measurements of activity, sleep, and movement. The actigraphs were initialized to record activity from 6:00 p.m. on day 1 and were worn by participants until they woke up after the night following the night of sleep deprivation.

Attentional Demands-Task (AD-Task). The Attentional Demands Task [77] is a computerized paradigm designed to assess selective, divided, and switching attention through the presentation of geometric figures (circle, square, triangle, diamond) in four colors (red, blue, green, yellow), each displayed centrally on a black screen for 250 ms, followed by a response window of up to 900 ms and variable inter-stimulus intervals of 250–1000 ms featuring a fixation cross. The task comprises 16 experimental blocks (eight selective and eight divided), preceded by two training blocks, with stimuli and response rules differing by condition: in selective attention blocks, participants consistently respond to a green circle with a single keypress, while in divided attention blocks, they respond to both a designated color and a designated shape, with stimulus-response mappings varying across blocks. The switching condition alternates blocks of selective and divided attention to measure cognitive costs associated with demand shifts, with six randomized sequences controlling for order effects. Across conditions, each block contains 60 trials, resulting in 240 stimuli per condition (174 nontargets and 66 targets), except for the switching condition, which totals 624 stimuli (468 nontargets and 156 targets). Performance is quantified by accuracy and reaction times, and the full procedure requires approximately 50 min.

### 4.3. Procedure

Both experiments were conducted at the Laboratory of Cognitive and Behavioral Sciences at the University of L’Aquila. In the first experiment, which investigated the influence of daily variations on attention, 32 participants were selected based on their Pittsburgh Sleep Quality Index (PSQI) scores and monitored using the Karolinska Sleep Diary (KSD) during the week of the experiment; none were smokers. The experimental protocol was carried out over two consecutive days. On the first day, participants completed a brief familiarization phase with the Attentional Demands Task (AD-Task) and filled out questionnaires assessing sleep quality, chronotype, and mood (rSCRAM). On the second day, they performed the full version of the AD-Task at four different times of the day (09:00, 12:00, 15:00, and 18:00).

The second experiment examined the effects of sleep deprivation on attention and involved 8 participants selected using the same PSQI-based criteria. Participants were monitored throughout the entire experimental week using both the Karolinska Sleep Diary (KSD) and actigraphs, which they wore continuously—including during the experimental days—until awakening on the morning following the night of total sleep deprivation. The testing schedule included sessions every three hours over two consecutive days, encompassing one night of total sleep deprivation for a total of 36 h of sustained wakefulness.

In both experiments, testing sessions were conducted in the same laboratory room, under controlled environmental conditions. The room was quiet, free from external distractions, and equipped with blackout-covered windows to minimize the influence of natural light. Constant artificial lighting conditions were maintained throughout all testing sessions. Participants were seated at individual workstations equipped with a computer (screen size: 35.4 × 19.9 cm). In the sleep deprivation experiment, mealtimes were standardized according to participants’ habitual eating schedules, with lunch and dinner occurring at the same times across the experimental protocol. During the total sleep deprivation period, participants were continuously monitored by trained research staff to ensure compliance with the protocol and maintenance of wakefulness. Although no neurophysiological recordings (e.g., EEG) were made, habitual daytime sleepiness was assessed at baseline using the Epworth Sleepiness Scale (ESS) by the research staff. Before each session, they completed the Karolinska Sleepiness Scale (KSS) and the Global Vigor and Affect Scale (G-VAS) to assess sleepiness, vigor, and affective state. Subsequently, they performed the AD-Task, presented in a randomized order, designed to measure selective attention, divided attention, and attentional switching. Each session lasted approximately 40 min. Throughout the experimental period, participants were instructed to abstain from excitatory substances such as caffeine, theine, and chocolate.

For performance assessment, accuracy was analyzed according to Signal Detection Theory (SDT) [95] computing hit rate (H), false alarm rate (FA), and sensitivity index (d′), with extreme value correction applied when necessary [96]. Reaction times (RTs) were used as an index of response speed.

### 4.4. Data Analysis

Descriptive statistics were performed on the characteristics of the sample (age, gender, schooling, occupation), on the chronotype (assessed through rSCRAM), on the indices of sleep quality and habitual sleepiness (measured through the completion of the rSCRAM, PSQI, ISI, and ESS questionnaires), and on the depressed and anxious mood (rSCRAM) of the participants. In addition, the averages of the scores obtained in the sleep diaries (KSDs) completed during the week of sleep monitoring (including experimental days) were calculated to consider sleep quality and restfulness. On the data from the aggregated analyzed actigraphs, averages of the variables of Sleep Onset, Final Awakening, Total Time in Bed (TIB), Total Sleep Time (TST), Sleep Onset Latency (SOL), Sleep Efficiency (SE), and Wake After Sleep Onset (WASO) were estimated to have a physiological index of the sleep characteristics of the participants in the week of experimentation.

To assess the variability of the indices of sleepiness, vigor, and affect, a repeated-measures ANOVA was performed in which TIME, understood as time of day, was included as a factor within the scores obtained on the KSS and GVAS (vigor and affect) questionnaires.

To assess variability in performance (HIT RATE. d’ and FA RATE) and speed (RTs) in the performance of the behavioral task AD-Task in selective and divided attention in the two different conditions of single demand and switching, at various times during the experimental day, a repeated-measures ANOVA was performed in which TIME (time of day), TYPE OF ATTENTIONAL DEMAND (selective/divided), and CONDITION (single demand/switching) were entered as within-subject factors. Post hoc comparisons were performed if statistical significance was reached (*p* < 0.05).

Whenever a statistically significant omnibus main effect or interaction was observed, post hoc pairwise comparisons were performed using Tukey-adjusted tests to account for multiple comparisons within each significant effect or interaction. Alongside *p*-values, partial eta-squared (η^2^p) was reported as a measure of effect size to facilitate the interpretation of the magnitude of the observed effects.

All analyses were conducted with the Jamovi 2.2.14 program, and the level of statistical significance was set at *p* < 0.05.

## 5. Conclusions

Taken with caution, these results may contribute to the idea that time of day and sleep deprivation can significantly modulate attentional performance. The task used demonstrates that selective and divided attention can exhibit different patterns of fluctuation throughout the day. Selective attention seems to be more vulnerable to diurnal fluctuations, with a marked decline in the afternoon, while divided attention seems to remain relatively stable, with a slight postprandial decline. The results also highlighted a discrepancy between subjective sleepiness and objective performance. Although participants reported no significant changes in perceived daytime sleepiness, their cognitive performance revealed some modulation based on time of day and sleep pressure. This discrepancy may be related to a partial dissociation between subjective perceptions of sleepiness and objective measures of attentional performance. However, alternative explanations cannot be ruled out, including the limited statistical power and sensitivity of the subjective measures used.

Under sleep deprivation, both selective and divided attention appear to be impaired, with slower reaction times and increased errors, especially in switching tasks.

From a practical perspective, these small findings highlight the importance of considering the effects of time of day and sleep deprivation in contexts where attentional efficiency is a key factor.

### 5.1. Practical Applications of the Attentional Demands Task

The Attentional Demands Task (AD-Task) is a useful tool in various contexts where attentional efficiency and cognitive flexibility are critical factors.

For example, in the workplace and organizational settings, the AD-Task could be used to assess the impact of work shifts, night work, and sleep deprivation on monitoring, multitasking, and adaptation to variable cognitive demands, providing useful information for optimizing schedules and preventing errors.

In high-responsibility and high-risk contexts, such as transportation, healthcare, and safety, the ability to distinguish between components of selective, divided, and switching attention allows for the identification of specific cognitive vulnerabilities related to the time of day or state of alertness, contributing to the development of targeted assessment and intervention protocols. Furthermore, in clinical and neuropsychological settings, the AD-Task could prove useful for monitoring attention deficits associated with sleep disorders, neurological or psychopathological conditions, as well as for evaluating the effectiveness of rehabilitative or pharmacological interventions. Beyond adult populations, the AD-Task may also be fruitfully applied in developmental and educational contexts. It offers a promising framework for investigating the maturation of attentional control across childhood and adolescence, as well as for examining the impact of environmental factors on attentional functioning. Notably, the task could be adapted to explore how exposure to digital technologies, multitasking media environments, and social media use influences sustained attention, attentional switching, and cognitive flexibility in younger populations. Such applications are especially relevant given current concerns about the effects of pervasive digital stimulation on attentional development and academic performance. Finally, the task’s sensitivity to daily and sleep pressure variations, combined with its capacity to simulate dynamic and ecologically valid attentional demands, supports its use as a powerful tool for assessing attention in real-world conditions. This makes the AD-Task particularly suitable for bridging experimental research and applied practice, contributing to a deeper understanding of how attentional processes operate in everyday life and across diverse populations.

### 5.2. Limitations

Despite the significant findings, several limitations should be acknowledged. First, although the sample sizes adopted in both phases were adequate for an initial experimental investigation, they may not fully represent the broader population, thus limiting the generalizability of the results. In particular, the relatively small sample size of the sleep deprivation experiment requires caution when interpreting higher-order interactions. Although the fully repeated-measures design increased statistical sensitivity by minimizing inter-individual variability, the limited number of participants inevitably reduced statistical power and the stability of parameter estimates. Consequently, the findings from the second phase should be considered preliminary and exploratory and warrant replication in larger independent samples before firm conclusions can be drawn. Furthermore, the biggest limitation concerns the absence of physiological measurements (e.g., EEG or body temperature), which compromises the possibility of correlating cognitive changes with underlying physiological changes. Another important limitation of our study is undoubtedly the lack of circadian physiological markers (such as melatonin secretion, core body temperature, or cortisol levels), which made it difficult to distinguish between endogenous circadian influences and masking effects related to environmental or behavioral factors. Therefore, the daily variations observed in attentional performance should be interpreted as effects related to the time of day rather than as the result of circadian modulation. In future studies, it is advisable to use behavioral measures alongside physiological assessments of the circadian phase to better characterize the relative contributions of circadian and homeostatic processes. About chronotype, it should be noted that although chronotype was assessed using the rSCRAM questionnaire, most participants scored in the intermediate range. This resulted in an insufficient representation of individuals who were clearly morning or evening types. Consequently, it was not possible in this study to directly examine the potential interaction between chronotype and time-of-day effects. Finally, sex/gender differences were not examined in either experiment due to the limited sample sizes. The first study was predominantly composed of female participants, while the second study included too few participants overall to allow for any reliable or balanced subgroup analyses.

## Figures and Tables

**Table 1:** Mean ± SD of the actigraphic sleep variables during the entire monitoring week. TSD = Total Sleep Deprivation. No sleep occurred during this period; therefore, actigraphic sleep variables could not be calculated. Sleep Onset and Final Awakening are reported as mean clock times (HH: MM). Remaining variables are reported as mean ± SD. Variables: Sleep Onset, Final Awakening, Total Time in Bed (TIB), Total Sleep Time (TST), Sleep Onset Latency (SOL), Sleep Efficiency (SE), and Wake After Sleep Onset (WASO).

	Sleep Onset Mean (HH:MM)	Final Awakening Mean (HH:MM)	TIB	TST	SOL	SE	WASO
DAY 1	1:47	06:59	08:12 ± 00:50	06:38 ± 01:16	25.75 ± 18.04	81.24 ± 11.41	55.25 ± 39.85
DAY 2	01:21	09:25	08:04 ± 01:24	06:50 ± 01:13	11.62 ± 9.07	84.68 ± 4.29	48.87 ± 34.41
DAY 3	01:11	06:50	08:39 ± 01:05	07:12 ± 00:56	8.5 ± 7.78	83.73 ± 9.49	51.62 ± 30.39
DAY 4	23:59	09:00	09:00 ± 01:19	07:47 ± 01:14	9.37 ± 13.45	87.95 ± 3.36	47.37 ± 20.65
DAY 5	00:26	09:46	09:20 ± 01:51	07:45 ± 01:58	8.87 ± 12.81	82.72 ± 7.99	59.37 ± 28.81
DAY 6	23:42	07:16	07:34 ± 01:17	06:32 ± 01:10	11.5 ± 20.17	87.68 ± 6.67	31.5 ± 8.45
DAY 7	TSD	TSD	TSD	TSD	TSD	TSD	TSD
DAY 8	22:08	06:01	07:52 ± 05:59	07:01 ± 06:15	2.71 ± 5.61	92.62 ± 2.99	43.14 ± 32.55

## Data Availability

The deidentified data supporting the findings of this study are available from the corresponding author upon reasonable request. The analyses were conducted using Jamovi (a graphical user interface); therefore, no reproducible code is available. Public sharing is not possible due to ethical and privacy restrictions. No preregistration was conducted for this study.

## Supplementary Materials

The following supporting information can be downloaded at: https://www.mdpi.com/article/10.3390/clockssleep8030041/s1.
