# Supplementary material for: Time-of-Day Variations and Effects of Total Sleep Deprivation on Attention Assessed with the Attentional Demands Task
Source: Clocks Sleep. 2026 Jul 2;8(3):41. doi: 10.3390/clockssleep8030041 (PMC13398160; doi:10.3390/clockssleep8030041)
Supplement: Supplementary file 1 [file clockssleep-08-00041-s001.zip › clockssleep-4357582-supplementary.pdf]

Descriptives – SLEEP QUALITY (KDS)				
	DAY 1	DAY 2	DAY 3	DAY 4
**N**	32	32	32	32
**Mean**	4.47	4.41	4.50	4.38
**Median**	4.75	4.75	4.50	4.38
**SD**	0.601	0.650	0.575	0.630

Table S1 - Sleep Quality assessment evaluated with KSD sleep diary

Descriptives - SLEEP RESTORATIVENESS (KSD)				
	DAY 1	DAY 2	DAY 3	DAY 4
**N**	32	32	32	32
**Mean**	3.82	3.71	3.56	3.35
**Median**	3.67	3.67	3.50	3.33
**SD**	0.750	0.820	0.773	0.708

Table S2 - Restorative Sleep assessment evaluated with KSD sleep diary

Descriptives - SLEEP QUALITY (KDS)							
	DAY 1	DAY 2	DAY 3	DAY 4	DAY 5	DAY 6	DAY 7
**N**	8	8	8	8	8	8	8
**Mean**	4.25	4.69	4.75	4.56	4.63	4.47	4.38
**Median**	4.50	4.75	5.00	4.75	5.00	4.75	4.75
**SD**	0.876	0.320	0.401	0.678	0.655	0.700	0.732

Table S3 - Sleep Quality assessment evaluated with KSD sleep diary

Descriptives - SLEEP RESTORATIVENESS (KSD)							
	DAY 1	DAY 2	DAY 3	DAY 4	DAY 5	DAY 6	DAY 7
**N**	8	8	8	8	8	8	8
**Mean**	3.83	4.04	3.87	4.04	4.42	3.58	3.71
**Median**	4.00	4.00	3.83	4.50	4.33	3.50	3.67
**SD**	0.667	0.603	0.665	1.16	0.527	0.792	0.744

Table S4 -Restorative Sleep assessment evaluated with KSD sleep diary
